# Plant health index as an anomaly detection tool for oil refinery processes

**DOI:** 10.1038/s41598-022-18824-2

**Published:** 2022-08-25

**Authors:** Fawaz S. Al-Anzi, Haitham M. S. Lababidi, Ghanima Al-Sharrah, Suad A. Al-Radwan, Ho Joon Seo

**Affiliations:** 1 Kuwait University, Kuwait City, Kuwait; 2 Australian College of Kuwait, Kuwait City, Kuwait; 3 BNF Technology, Daejeon, South Korea

**Keywords:** Chemistry, Engineering, Mathematics and computing

## Abstract

Early detection of significant abnormal changes is highly desirable for oil refinery processes, which consist of sophisticated unit operations handling hazardous and flammable inventories and operating at high temperature and pressure. Close monitoring and anomaly detection are vital for avoiding major accidents and losses and enable intervention before failure occurrence. A new big data analytics tool called Plant Health Index (PHI) is proposed in this work. PHI is a statistical anomaly detection software that trains its model using online normal plant operation, then uses statistical analytics to detect anomalies. For detecting the anomalies, a combined method of multivariate analysis of residuals and nonparametric models of the process is employed. The methodology provides a structured representation of the plant variables to ease the detection of problems along with the detection of operation changes of the system. The PHI system has been tested on a hydrotreating units in a refinery, which consists of catalytic reactors and separators. The current implementation tagged 170 process variables and proved effective in capturing the normal operational conditions of the plant. When placed online, PHI was able of detecting anomalies that are difficult to detect using the control system and before being detected by the alarm system.

## Introduction

Oil refineries are among the most complicated dynamical structures, requiring smooth, effective and safe operation to continuously produce high quality products at competitive costs. Extremely sophisticated surveillance systems are necessary, with early identification of plant malfunctions and anomalous behavior. Machine learning algorithms can be effectively used to discover anomalies based on online and historical data, which can lead to system health monitoring. When studying real-world data sets, knowing which examples stand out as being different from all others is a common requirement. Anomalies are these types of events, and the purpose of outlier detection or anomaly detection is to find all of them using online operational data^1^.

The petroleum sector has progressed into a highly regulated industry with operational safety and security as core goals. Almost all installed equipment in modern refineries has sensors that monitor their activity and remote-controlled actuators to operate on them in order to manage the operational profile, avoid unwanted events, and avoid catastrophic failures. The physical integrity of oil and gas plants are strictly protected through multilayers of control and alarm systems that react to unusual circumstances. Anomaly detection is important because anomalies in data can lead to significant actionable information in a range of application fields^1^. The capacity to execute on the environment to appropriately respond, prevent, or remedy the situations associated with such unique information gives the decision maker the ability to identify it correctly^2^.

Another important consideration in process industries, such as oil refineries, is handling of large quantities of hazardous and inflammable materials, which flow at high rates (tons per hour), high temperatures (hundreds of degrees Celsius), and power (in megawatts)^3^. Thousands of personnel and millions of dollars are on the line every second, as a single small flaw or error can cause significant harm to the entire plant and its workers, as well as income losses. As a result, the upmost concern of the industrial plant management is to ensure continuous safety, process efficiency, long-term durability, and scheduled (vs. unscheduled) downtime. Distributed control systems (DCS) and supervisory control and data acquisition (SCADA) systems are commonly used for continuous monitoring and control of equipment and unit operations, such as pumps, compressors, separators, boilers, heat exchangers and catalytic reactors. Variables that are generally measured and transmitted as signals are temperature, flowrate, level, pressure, and vibration. With hundreds or thousands of monitoring sensors used throughout the process plant, keeping track of whether they are working properly or not is very time consuming and labor expensive^4^.

Advanced computing technologies and availability of cheaper storage medium enabled process industries to accumulate huge amount of time-stamped data that are stored as records of all measurements for the past months or years. Process “historians” available in the DCS systems provide useful statistical and visualization tools for processing and comparing previous operational trends. However, the predictive abilities of such tools are very limited and operators and technicians, who are usually overworked, rely on their intuition and experience particularly when it comes to finding useful patterns in the massive data and deducing expected future deviations and anomalies that may result in unanticipated damages and losses. As a result, effective models for monitoring and identifying abnormalities or anomalies based on various sensors and historical data connected to plant operations are becoming increasingly important and on demand. Here, data mining and machine learning approaches may prove effective in solving the challenges faced by oil and gas industries.

**Figure 1:**
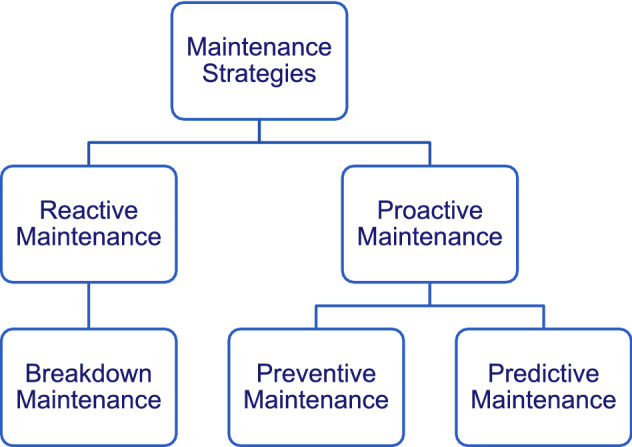
Maintenance strategies.

This paper attempts to explore the utilization of Big Data analytics in oil refinery maintenance processes context. Maintenance of plant equipment and sensors is crucial for normal plant operation. The objective of maintenance support systems is to identify degradation in equipment or component and rectify or replace those to keep up original functions. Maintenance strategies can be categorized under *reactive* or *proactive* maintenance (see Fig. 1). Conventional strategies for maintenance are usually based on reactive or corrective, under which equipment is replaced or if and only if it has damaged or undergo severe performance degradation. This ‘‘run to failure” strategy, known as *breakdown maintenance*, is still broadly appropriated for equipment whose failure will not impact operations and can be quickly and easily come back to service or whose failure timing and modes do not exhibit a significant statistical pattern. However, the consequences of some failures may be far-reaching and expensive. These failures can result in serious operational difficulties or even shut down the plant causing serious economic losses. For such circumstances, *proactive maintenance* strategies (see Fig. 1) are necessary to avoid serious failures. A commonly used proactive strategy is *preventive maintenance* which is based on analyzing the equipment failure history, and consequently, a maintenance program is planned and conducted to fix equipment before failing. However, in some cases, preventive maintenance may be costly or even wasteful. A new maintenance strategy was developed to reduce the maintenance expenditure. In this strategy, equipment conditions are regularly monitored until equipment starts to produce evidence of developing failure or deteriorating performance. To prevent failures in equipment, ‘‘just-in-time” maintenance is performed, known as *predictive maintenance* or *condition-based maintenance* (CBM).

Condition-based maintenance (CBM) can reduce cost expenditure by reducing unnecessary and time-consuming maintenance activities and reducing human errors. One of the essential methodologies in CBM is to predict an anticipated normal state compared to a measured observation. If the difference between the expected state and the observed state increases, one can suspect an anomaly in the system. There are two types of models that are used in predicting normal states in the framework of CBM. The model is derived from basic physical principles for the first type, while for the second one, the model is inferred from historical observations^5^. This research focused on the latter model type, which is sometimes considered an empirical model based on statistical analytics. Empirical models are more practical for the following reasons^6^:

- Context-free applicability: This indicates that without any detailed system knowledge, the core of an empirical model can be easily employed in any form of system modeling.
- Configuration flexibility: This indicates that it can be flexibly monitored and identified the system boundary.
- Customization adaptability: This indicates that ageing modeling is relatively easy to customize a reference or baseline performance.

While various pilot-scale models utilizing empirical models have been employed at the worksites in nuclear facilities, these models should not be marked as an appreciated one than conventional physical models concerning accuracy. As a result of the inspection done to recognize the bottleneck in former efforts^7^, we have identified the necessity for:

- A novel parameter grouping strategy like an empirical model can adopt physical knowledge on a system to some extent
- Predicting plant health status using statistical signatures for process anomalies.

This research proposes and implements an enhanced framework including the suggested strategy and providing a software system with a graphical user interface illustrating the functions of the strategy. Plant Health Index (PHI) Solutions have been proven in predicting the operational performance (health) of nuclear, power, and desalination plants. Following the implementation and assessment of PHI in these plants, the objective is to prove the applicability of PHI in other process industries. In the current work, the applicability of PHI in refinery operation is investigated. Hence, the primary objectives are:

- Evaluate the applicability of PHI in selected refinery operations.
- Assess PHI in analyzing the plant behavior and evaluate the reliability of its predictions.

The research work also aims to answer the following queries:

- What are data characteristics specifically for the refinery processes?
- What cost savings does PHI bring to the selected process?
- How to identify and optimize the health condition of the selected process using big data analytics and data mining techniques?

## Background

While operating a plant, if the outputs are good and there are no warnings, it does not mean that the plant is healthy. It just means that the current results are decent while unrecognized problems may be developing in the plant. Therefore, there could be potential problems or abnormalities in the health of the plant. However, since there is no knowledge on the status or health of the plant, there is always a possibility of risk of failure/trip without early alerts.

**Figure 2:**
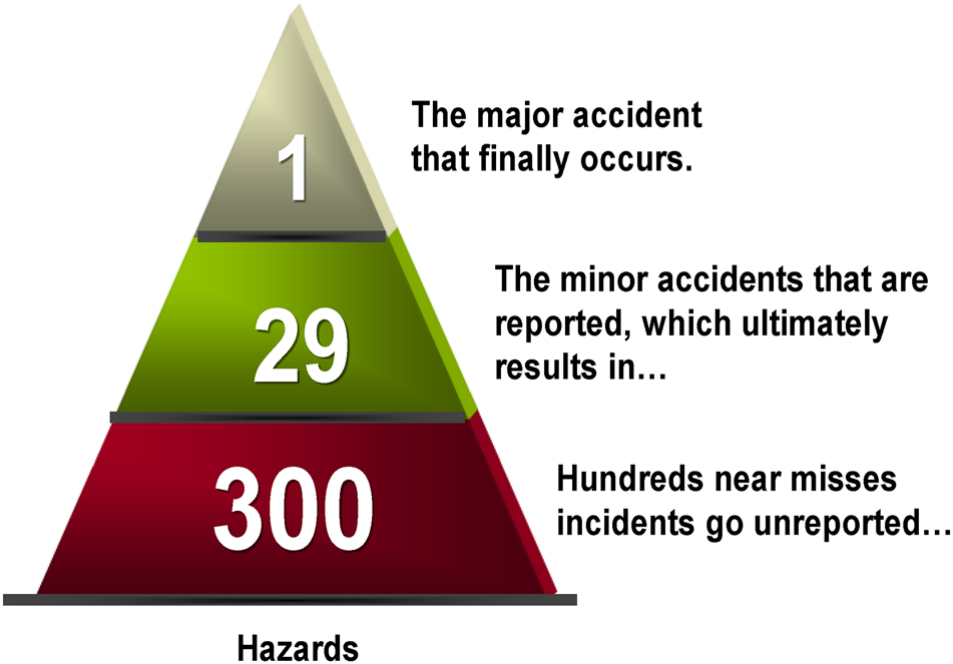
Heinrich Law (300-29-1 Model).

According to Heinrich’s Law (see Fig. 2), for every major accident in the workplace, there are 29 minor accidents and 300 near miss incidents^8^. Because most accidents have the same root causes, addressing more commonplace near-miss accidents can prevent minor and major accidents^9,10^. Therefore, what if we monitor not only 29 minor accidents but all 300 incidents? In fact, the main concept of the Plant Health Index (PHI) approach proposed in this study is to monitor the operation of a chemical plant, derive symptoms, and predict the occurrence of all incidents.

**Figure 3:**
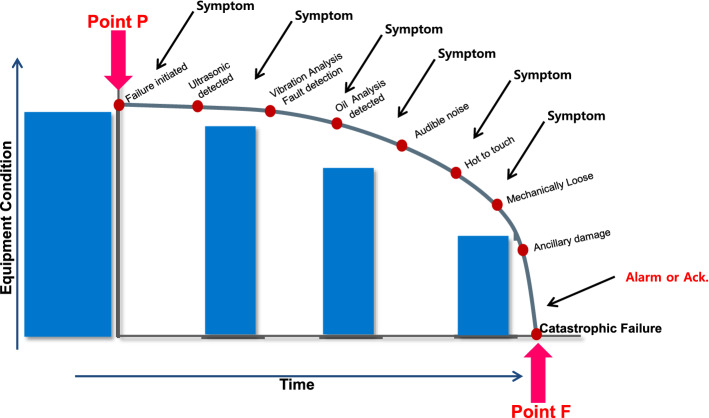
Equipment failure trajectory.

Knowing that failures, whether minor or catastrophic, are preceded by symptoms that indicate deterioration in equipment or process conditions (see Fig. 3), the question is: How can a failure be detected early enough to provide time to plan and schedule work without panic or reactivity?

The specific objective is to adapt the proposed PHI software to refinery operation. The implemented PHI system is expected to evaluate a health index that would reflect the operational performance of the selected refinery section. When displayed, the numeric value of the index could be easily interpreted by technical and non-technical personnel for a better understanding of the status of the plant. Technological problems that are expected to be solved by implementing PHI as a big data analytics tool include:

- Preventive and predictive maintenance policy for refinery operation.
- Reducing downtime and reducing non-operational and idle time in refinery operation.
- More effective logistic operations of spare parts and upgrades of a refinery’s back office.
- Bringing in cost benefits by implementing the solution in refineries.

The methodology that will be followed in reaching the specified objectives consists of the following steps:

1. Designing and installing Plant Health Index solution in a selected refinery process.
2. Analyzing the output and prediction performance of PHI.
3. Introducing new algorithms for adapting PHI to specific refinery big data anomalies.
4. Evaluate the practical application of the algorithm for refinery operation.
5. Involve experienced operators in analyzing and commenting on the diagnostic performance of PHI.
6. Evaluate the contribution of the system as a predictive refinery big data diagnostics solution.

The optimization of industrial processes has received a lot of attention in past few decades. Several fields of study in the activity tracking and fault-detection and isolation (FDI) domains have sprung up because of this. Model-based, data-driven, and hybrid techniques are the three types of extant methodologies. Model-based techniques that use first-principle models necessitate a high level of a priori information about the systems being studied. As a result, applying them to big structures can be difficult, especially if portions of these structures are unclear. Data-driven techniques classify a system's operation based on accessible estimation of activities. In most cases, training set should be needed to describe the conventional functioning of the system so that defects can be recognized as departures from the learnt nominal process characteristics. Additional data indicating the process's characteristic behavior while it is vulnerable to failures is frequently required for fault isolation utilizing data-driven techniques. Authentic benchmark examples are necessary to compare the performance of various FDI approaches. Since Downs and Vogel's first release in 1993^11^, the Tennessee Eastman method (TEP) has been widely employed for this purpose. It gives a benchmark scenario that may be utilized to look at most of the problems that continuous processes can cause. Reinartz et al.^12^ contributed a large reference dataset, which includes repeated simulations of normal and faulty process data, as well as supplementary observations and different significance levels for all process disruptions.

In recent years, multimode operation monitoring has attracted a lot of interest in academic and commercial. In general speaking, all the factors that may be monitored are used to supervise an operation. Insignificant factors, on the other hand, may reduce the effectiveness of monitoring due to over-fitting and significantly raise the online computing overhead. It is necessary to choose the right parameters to be monitored that are strongly linked to these problems to track the problems that may compromise safety, reliability and product quality. Wu et al.^13^ conducted a study for exploring the effects of multimode process monitoring with parameter selection. For selecting variables, a KLD (Kullback–Leibler divergence) based approach is given, with the goal of selecting the most useful variables concerning the defects in question. Variable selection aids in the development of a high-performance model with a low risk of overfitting, as well as improving the interpretation for accurate problem diagnosis. In addition, they proposed and presented a new detection index named moving window-negative log likelihood probability (MWNLLP) for online monitoring. They employed both a numerical example and the TEP (Tennessee Eastman process) for demonstrating the usefulness of the suggested method^13^.

Technologically advanced production plants require process monitoring tools that are not only highly intelligent, but also most reliable and secure. Substantial advances have been achieved in the last decades on data-driven activity monitoring approaches, with a huge variety of parameters monitored and saved, and most of these have been successfully deployed to monitor diverse processes. The work conducted by Cheng et al.^14^ employed gated recurrent unit (GRU) for formulating variational recurrent autoencoder (VRAE) since its performance is comparable to that of LSTM (long short-term memory), but it is computationally more efficient. Furthermore, they introduced a monitoring metric, the negative variational score (NVS) for process industry problem identification. Whereas all previous approaches rely on reconstruction error to detect anomalies, the suggested NVS considers both reconstruction error and similarities among the prior and posterior probabilistic distribution. They evaluated the effectiveness of the proposed model in detecting the faults using standard statistical and artificial intelligence-based approaches on both simple nonlinear simulation and the benchmark TEP^14^.

Radcliffe and Reklaitis^15^ reported a quality control and monitoring method for a pharmaceutical production systems. In a related study, a hierarchical Bayesian technique has been utilized to build hybrid models that relate inline picture capture information to outputs^21^. Explanatory factors arising directly from the picture data are given a functional shape by known physics, whilst unknown effects arising from the interaction of process circumstances, ink qualities, and particle features are represented in hierarchical manner. It was feasible to completely apply the resources included in the historical information, that comprises of many understandings of interchangeable datasets stratified across various levels, by simulating the interactions of these unknown consequences in a hierarchical regression analysis^16^.

## The health index concept

Most systems have their own design or optimized conditions in terms of safety and efficiency. However, off-design conditions are likely to harm operational stability. For instance, if a safety parameter starts to deviate from a design condition and is approaching the process limits, it means the possibility of failure is getting higher. Likewise, if a performance parameter is getting more deviated from a design condition, the efficiency of a plant will be going down^17^. Therefore, the deviation from a design condition can be a good reference in defining the system’s health status.

**Figure 4:**
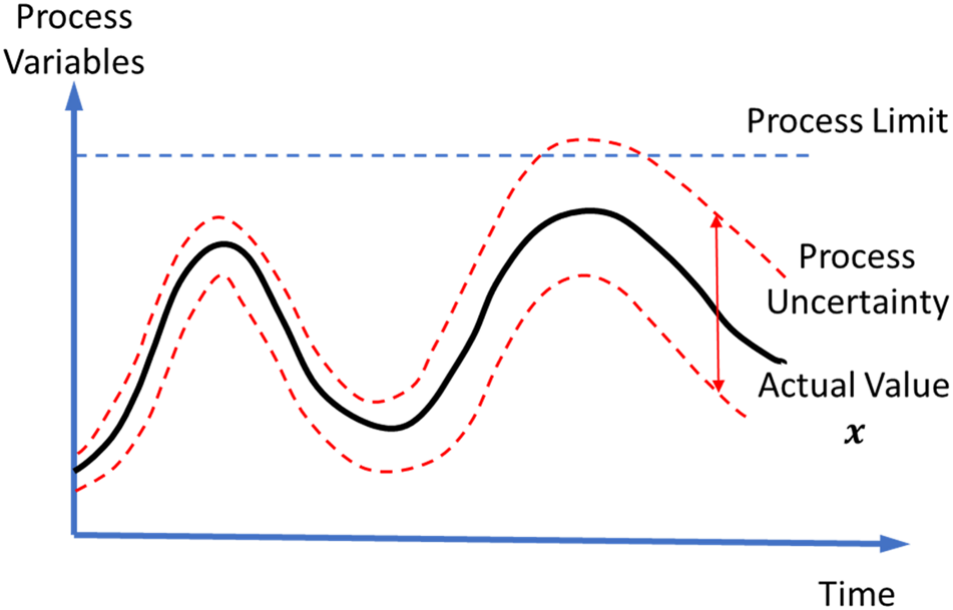
Definition of Process Uncertainty.

Process uncertainty is another metric to define health status of a system. It shows whether the behavior of a process component is as expected or not. As shown in Fig. 4, if the process uncertainty is narrow, the component is close to the expected behavior, whereas wider ranges indicate malfunctions in the component or the system. Process uncertainty is thus characterized by the health or illness of the process, which is related to the limits of the operational conditions of the process. By monitoring the process uncertainty, it is possible to detect abnormal behaviors even though the system is operating with enough margins to process limits. This feature, referred to as early detection capability, can help operators deal with abnormal procedures before failures and eventually support condition-based maintenance^8^.

### Evaluation of Health Index

**Figure 5:**
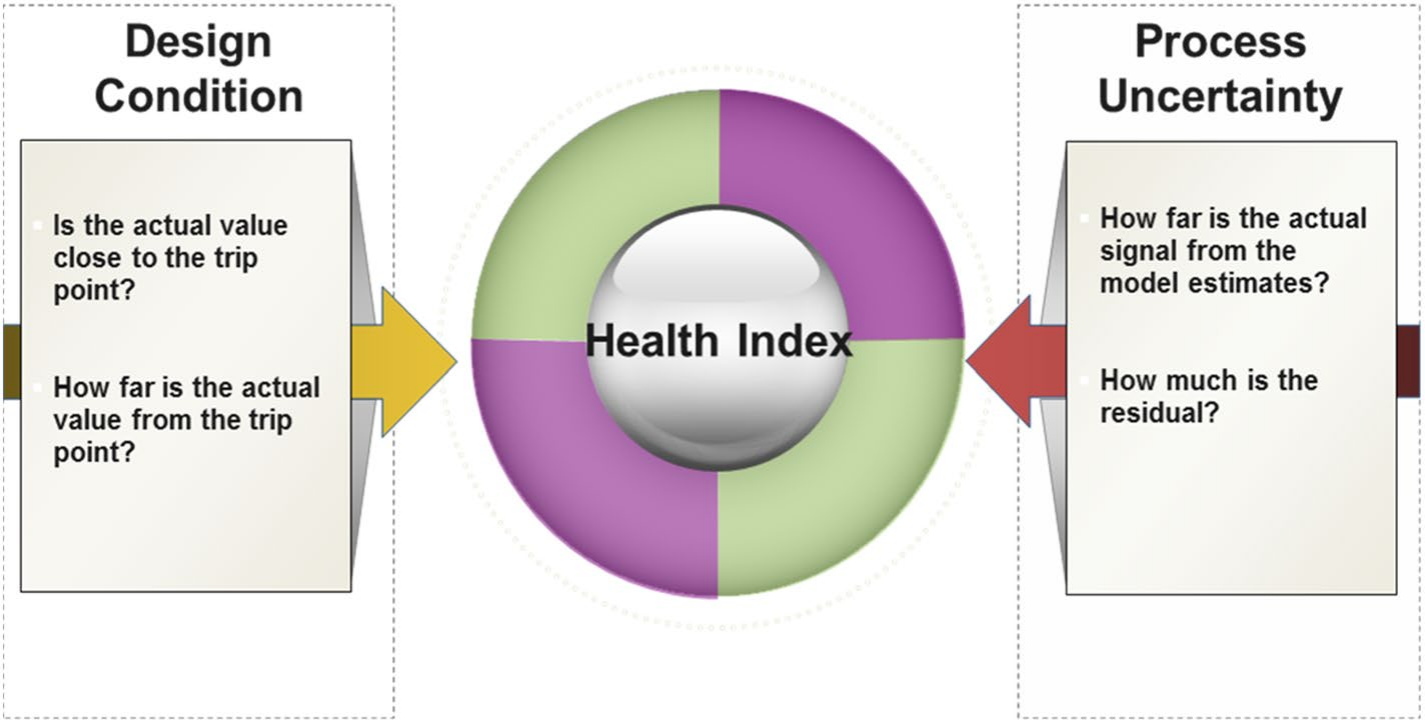
Health Index accounts for deviations of actual measurement from design and nominal conditions.

The proposed health index quantifies the overall health status of a process plant by aggregating individual indicators calculated for each component based on a functional success tree, tagged with its weight of importance. The health index of a component can be obtained by combining the health status, representing deviations from design conditions, and process uncertainty, related to deviations from model estimates^18^. Figure 5 illustrates the concept of the health index.

**Figure 6:**
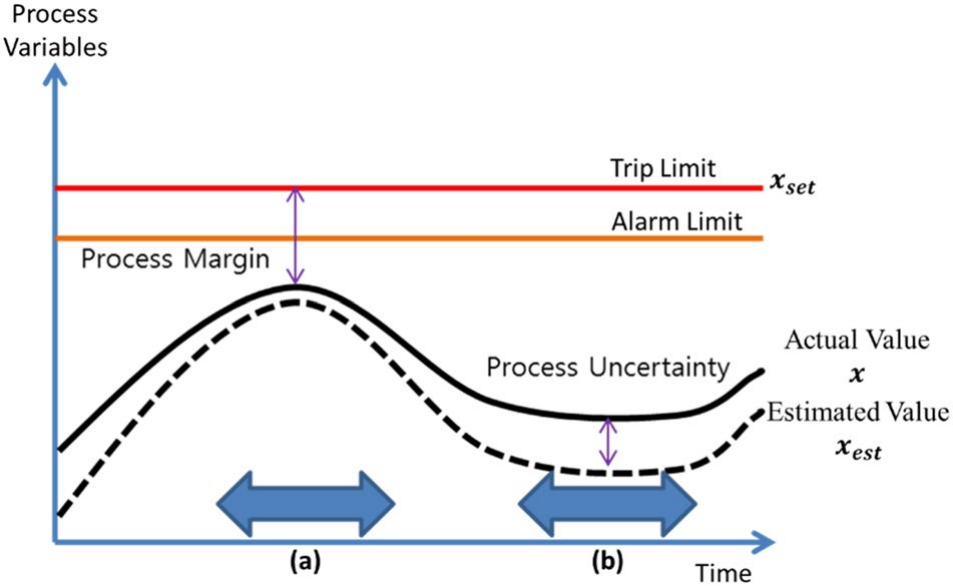
Process margin and process uncertainty. Plant health: (**a**) less uncertain but less margin, and (**b**) more margin but more uncertain.

As shown in Fig. 6, *Process margin* is defined as the difference between an alarm/trip and an operational condition, while *process uncertainty* (or healthiness) is defined as the residual between an anticipated normal condition and an operational condition. The main concept behind calculating the health index is providing an early warning by observing abnormal process uncertainty earlier than those of process margin.

**Figure 7:**
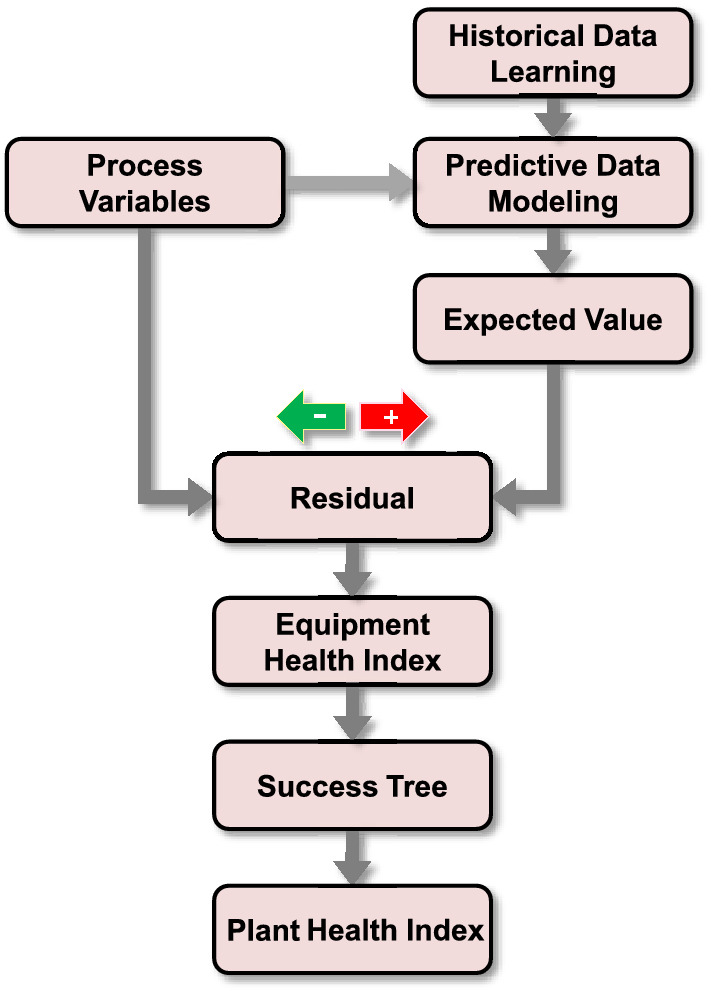
Procedure used in calculating the plant health index ^19^.

The procedure used in calculating the overall health index is shown in Fig. 7. Historical data is used in developing the empirical predictive model, which is used to estimate the desired values $x_{est}$ (see Fig. 6), while online process data (*x*) is used in calculating the residuals needed to determine the different components of the indices.

The setpoints $\left( {x_{set} } \right)$ are defined as the trip- or alarm-setpoints for safety-related parameters. These setpoints are usually set as design setpoints but can be modified to other suitable values. In fact, for the current implementation, the setpoints were selected based on the historical limits. Moreover, setpoints related to performance parameters are set as allowable limits derived from economic considerations. Another important monitoring references are the *nominal conditions* (*µ*), which are normally defined as the design conditions of process components.

The upper and lower limits of the health index are set as follows; when the current operating conditions (*x*) approach the nominal conditions (*µ*), the health index should be 1.0, while when the operating conditions approach the setpoints $\left( {x_{set} } \right)$, the health index should be 0.0. Thus, *µ* and $x_{set}$, are the two boundaries that define the best and worst plant health conditions, and the health index distribution between the two boundaries $\left( {HI_{margin} } \right)$ can be defined as:

1 $$HI_{margin} = 1 - \left( {\frac{x - \mu }{{x_{set} - \mu }}} \right)^{2}$$

The process uncertainty component of the health index $\left( {HI_{uncertain} } \right)$, defined by Eq. (2), represents the residual between the current condition and the estimated condition $\left( {x_{est} } \right)$. Its value is 1.0 if the current operating conditions are the same as the model estimates, which means that the residual is minimal (→ 0.0). On the other hand, if the actual operating condition is far from the model estimate, the residual is very large (→ ∞) and the value of the index approaches 0.0.

2 $$HI_{uncertain} = e^{{ - \left( {x - x_{est} } \right)}}$$

The overall health index $\left( {HI_{Overall} } \right)$ for a given component can be represented as combination of the margin and uncertainty health indices:

3 $$HI_{Overall} = HI_{margin} \times HI_{uncertain}$$

while $HI_{Overall}$ represents the health index of an individual component (child index), the health index at a system-level (or group index) can be obtained by conditional combination of the indices evaluated for individual components. The system under study is divided into *N* sub-systems (groups), which are considered as “parents”, and each sub-system consists of *M* components. Hence, the $HI_{Overall}$ index given by Eq. (3) will be referred to as $HI_{i,j}$, which refers to the *j*th component of the *i*th group.

The combination method is based on the *minmax* algorithm, which maximizes the minimum gain. The steps followed in determining the group and system-level health indices are summarized as follows:

1. Defined an initial *Compensation Factor* $\left( {CompFactor} \right)$ in the range of 0.1 to 1.2. This factor is to offset variability in very small sample sizes.
2. For each group (parent) find the top two maximum indices (*HI*_max,1_ and *HI*_max,2_) and the lowest two minimum values of indices (*HI*_min,1_ and *HI*_min,2_) for all components of the group.
3. Calculate the average value as:
  4 $$MM_{Avg} = \frac{1}{4}\left( {\left( {HI_{{{\text{max}},1}} - HI_{{{\text{min}},1}} } \right) + \left( {HI_{{{\text{max}},1}} - HI_{{{\text{min}},2}} } \right) + \left( {HI_{{{\text{max}},2}} - HI_{{{\text{min}},1}} } \right) + \left( {HI_{{{\text{max}},2}} - HI_{{{\text{min}},2}} } \right)} \right)$$
4. Calculate a new Compensation Factor:
  5 $$NewCompFactor = CompFactor - \frac{2}{100}MM_{Avg}$$
5. Calculate the average of the index values of all components of a given group:
  6 $$IndexAvg_{i} = \frac{1}{M}\mathop \sum \limits_{j = 1}^{M} HI_{i,j}$$
  where *i* indicates the group (parent) and *j* indicates the component (child).
6. Calculate average index using the new Compensation Factor:
  7 $$IndexAvgComp_{i} = IndexAvg_{i} \times NewCompFactor$$
7. Set the value of the index for parent *i* as:
  8 $$\begin{aligned} & IF\;\;IndexAvgComp_{i} < HI_{{{\text{min}},1}} \\ & \quad \quad Index_{i} = HI_{{{\text{min}},1}} \\ & Else \\ & \quad \quad Index_{i} = IndexAvgComp_{i} \\ \end{aligned}$$

The overall value of the health index can be customized based on the preferences of stakeholders. For instance, the PHI may consider safety or performance separately or combined in one index. Moreover, the health index calculation, the weighting factors of each term, and the combination of system-level health indexes can be changed depending on plant and operation situations.

## Plant health index development

This section focuses on developing a grouping technique and the framework to monitor the health status of a chemical plant. Each monitoring module calculates the health index and integrates it into the overall health index to present the plant’s health status^18^. This methodology will be applied to the selected section of the refinery.

Research activities carried out in this study focused on statistical learning strategies for supporting condition-based maintenance (CBM). The proposed system is composed of a training mode and an execution mode. First, an empirical model is developed using the data collected from normal working conditions in the training mode. In contrast, execution mode involves deciding on an anomaly band by inspecting input operational data and studying its deviation from the modeling output from the training mode.

The Health Index can be envisioned to be developed using the following steps:

- *Step I*: Design Condition Monitoring.
  The first health index is characterized by the deviation between a design condition and an actual condition of process variables (*HI*_*margin*_, Eq. (1)). Thus, health indices are quantified for both safety- and efficiency-related variables.
- *Step II*: Process Uncertainty Monitoring.
  Process uncertainty is based on the outputs of the empirical model. Residual is assigned by the difference between the expected value that a model estimates and an actual value (*HI*_uncertain_, Eq. (2)). Based on this residual, a health index is obtained.
- *Step III*: Overall Health Index.
  The overall health index of a component (*HI*_*Overall*_, Eq. (3)) is represented by the combination of a health index jointly coupled with design condition and residual size.
- *Step VI*: Software of Health Index Monitoring.
  Operation supporting system is developed by using the process pattern recognition technology proposed in this research. The system is designed to provide a graphical user interface consisting of the main display, success tree display, trends display, counseling display, trainer, and runtime. Using the success tree display, the operators should be able to configure the tree and the weight of each node. Trends display is designed to include actual values and model estimates of process variables. Counseling display is provided to support operators in diagnosing the detected faults. Finally, operators can decide sampling methods, grouping options, and kernel optimization methods in the trainer and runtime module.

### PHI deployment approach

The following practical steps were followed in deploying the Plant Health Index (PHI) system for the selected Refinery Hydrotreating unit:

- Identify a suitable subsystem for system Implementation.
- Install PHI solution for the selected subsystem.
- Provide system configuration range.
- Define and provide the interface connected to the system, which is an industry-standard, such as OPC.
- Retrieve historical data for a minimum of 6 months.
- Assign engineers to evaluate the system performance.
- Analyze the output of the solution.
- Arrange site experts to comment on the advantages of the solutions.
- Evaluate the practical application of the algorithm for the selected process.
- Evaluate the contribution of the system as a predictive diagnostics solution.

**Figure 8:**
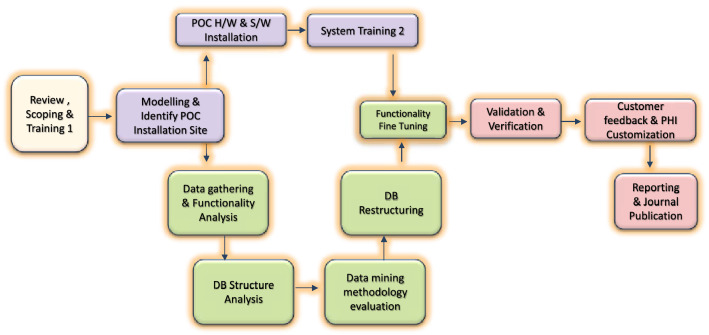
PHI deployment approach.

The approach outlined above is represented in the flowchart shown in Fig. 8.

## Implementation of PHI

PHI has been designed to capture and assess the condition of equipment during its life cycle. Thus, it may be utilized in data-driven condition-based maintenance and helps in predicting failures and malfunctions^20^.

### Data collection and preprossing

Data Acquisition refers to collection of historical data for a long duration for training a predictive model under normal operating conditions. It is preferable that collected data contains various operating modes and may also include abnormal conditions and operational variations that result from, for example, aging of equipment, fouling, and catalyst deactivation.

The training datasets are collected in real-time directly from the sensors associated with the plant components. The datasets capture the three operational modes; i.e. startup mode, normal operating mode, and shutdown mode. These modes can be subdivided into more detailed modes in some circumstances.

Although the parameters possess a strong correlation, the time lag appears among them may lead to the inability to extract the relationship. The explanation for the time delay in parameters with physical relationships is that it takes time to reach a steady-state once certain changes occur and migrate from one portion to another. However, if the parameters have a strong association, if they change over time, the correlation coefficient may be modest, resulting in errors during the grouping procedure. We employed a dynamic window for sampling which examines the temporal lag among parameters to aid in the effective grouping of variables with a strong link.

The time lag was dealt with using cross correlation. For a delay duration of $t_{d}$, Eq. (9) defines the coefficient for cross correlation between two parameters $A$ ($a_{0}$, $a_{1} , \ldots , a_{M}$) and $B$ ($b_{0}$, $b_{1} , \ldots , b_{M}$)^21^. The averages of $A$ and $B$ are $\mu_{A}$ and $\mu_{A}$, respectively.

9 $${\upgamma }_{AB} \left( {t_{d} } \right) = \frac{{\mathop \sum \nolimits_{i = 0}^{M - 1} \left( {a_{i} - {\upmu }_{A} } \right)*\left( {b_{{i - t_{d} }} - {\upmu }_{B} } \right)}}{{\sqrt {\mathop \sum \nolimits_{i = 0}^{M - 1} \left( {a_{i} - \mu_{A} } \right)^{2} } \sqrt {\mathop \sum \nolimits_{i = 0}^{M - 1} \left( {b_{{i - t_{d} }} - \mu_{B} } \right)^{2} } }}$$

Grouping parameters aims to remove elements that don't provide meaningful data and to limit the number of parameters needed to adequately observe a component. The correlation coefficient employed as a reference for this grouping procedure is calculated for each pair of variables using Eq. (10), and if it exceeds a specified threshold, the variable is included in the training set; otherwise, it is discarded^21^.

10 $$\rho_{AB} = \frac{1}{M}\mathop \sum \limits_{i = 0}^{M - 1} \left( {\frac{{a_{i} - {\upmu }_{A} }}{{{\upsigma }_{A} }}} \right)\left( {\frac{{b_{i} - {\upmu }_{B} }}{{{\upsigma }_{B} }}} \right)$$

where $\rho_{AB}$ is the correlation coefficient among $A$ and $B$, and $\sigma_{A}$ and $\sigma_{B}$ are their standard deviations.

There are three possible ways to group the parameters: Relational grouping (tags with the same patterns are grouped together), Manual grouping (each group possesses all of the tags), and Success Tree based grouping. The cut-off value of the correlation coefficients is known as group sensitivity. The grouping will become more precise if the group sensitivity is larger. When data is compressed during grouping, the Group Resolution (Shrink) feature is employed. If a tag has 1000 samples and the compression ratio is 100, the samples will be compressed to 100 and the missing information will be filled in by the Grid Size. Major significance of compression includes reduced data storage, data transfer time, and communication bandwidth. Time-series datasets frequently grow to terabytes and beyond. It is necessary to compress the datasets collected for attaining most effective model while preserving available resources.

Preprocessing of collected data is indispensable to ensure the accuracy of the developed empirical models, which are sensitive to noise and outliers. The selection of the sampling rate is also crucial, mainly because for the oil refinery processes the sampling rate (measurement frequency) is much faster than the process dynamics. In the current implementation, low pass frequency filtering with Fourier analysis was used to eliminate outliers, a 10 min sampling rate was selected, and the compression rate (Group resolution or shrink) was set at 1000. Moreover, Kalman filter was applied to ensure robust noise distribution of collected data^5^. Another important preprocessing step is *grouping*. First, the useful information of the variables is grouped together. It helps to remove redundant variables that do not have useful information. It also reduces the number of variables required for monitoring the plant properly. Finally, the available information must be appropriately compressed via the transformation of high-dimensional data sets into low-dimensional features with minimal loss of class separability^21^. The maximum tags per group is limited to 51 in this simulation and success tree-based grouping is used in most of the cases. The minimum value of the correlation coefficient, $\rho$ is set to 0.20 and the group sensitivity was set to 0.90. Higher the group sensitivity will be more accurate the grouping.

### Kernel regression

Kernel regression is a well-known non-parametric method for estimating a random variable's conditional expectation^22–25^. The goal is to discover a non-linear relationship of the two random variables. When dealing with data that has a skewed distribution, the kernel regression is a good choice to use. This model determines the value of the parameter by estimating the exemplar observation and weighted average of historical data. The Kernel function is considered as weights in kernel regression. It is a symmetric, continuous, and limited real function that integrate to 1. The kernel function can't have a negative value. The Nardaraya–Watson estimator given by Eq. (11) is the most concise way to express kernel regression estimating $y$ with respect to the input $x$^21,23,24^.

11 $$\hat{y} = \frac{{\mathop \sum \nolimits_{i = 1}^{n} \left[ {K\left( {X_{i} - x} \right)Y_{i} } \right]}}{{\mathop \sum \nolimits_{i = 1}^{n} K\left( {X_{i} - x} \right)}}$$

The selection of appropriate kernel for the situation is limited by practical and theoretical concerns. Reported Kernels are Epanechnikov, Gaussian, Quartic (biweight), Tricube (triweight), Uniform, Triangular, Cosine, Logistics, and Sigmoid ^25^. In the current implementation of PHI, three types of the kernel regression are provided: Uniform, Triangular, and Gaussian, which are defined as:

- Uniform Kernel (Rectangular window): $K\left( x \right) = \frac{1}{2}; where \left| x \right| \le 1$
- Triangular Kernel (Triangular window): $K\left( x \right) = 1 - \left| x \right|; where \left| x \right| \le 1$
- Gaussian Kernel: $$K\left( x \right) = \frac{1}{{\sqrt {2\pi } }}e^{{ - \frac{{x^{2} }}{2}}}$$

The default is the Gaussian kernel which proved to be the most effective kernel for the current implementation.

### Simulation of PHI

**Figure 9:**
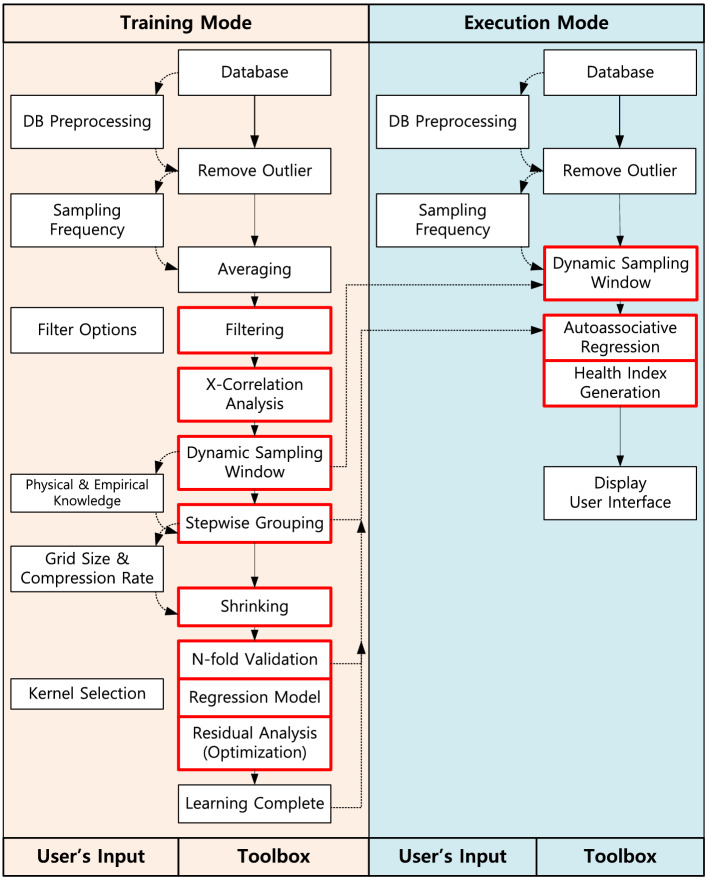
Algorithms of the PHI ^26^.

PHI monitors plant signals, derives actual values of operational variables, compares actual values with expected values predicted using empirical models, and quantifies deviations between actual and expected values. Before positioning it to monitor plant operation, PHI should be first trained to predict the normal operating conditions of a process. Developing the empirical predictive model is based on a statistical learning technique consisting of an “execution mode” and a “training mode.” Methods and algorithms used in both modes of the PHI system are shown in Fig. 9.

In the training mode, statistical methods are used to train the model using past operating data. The system identifies possible anomalies in operation for the execution mode by inspecting the discrepancies between values predicted by the empirical model and actual online measurements. For example, if a current operating condition approaches the normal condition, the health index is 100%. As opposed, if an operating condition approaches the alarm set point, the health index will be 0%. On the other hand, and in terms of process uncertainty, the health index is characterized by the residual deviations; the health index is 100% if a current operating condition is the same as the model estimate (i.e., the residual is 0.0), and is 0% if the operating conditions are far enough from the model estimate (i.e., residual is infinity). The overall plant index is a combination of the above two health indices. Details of the method are presented in^21^ and^26^ and presented as an improved statistical learning framework described below.

**Figure 10:**
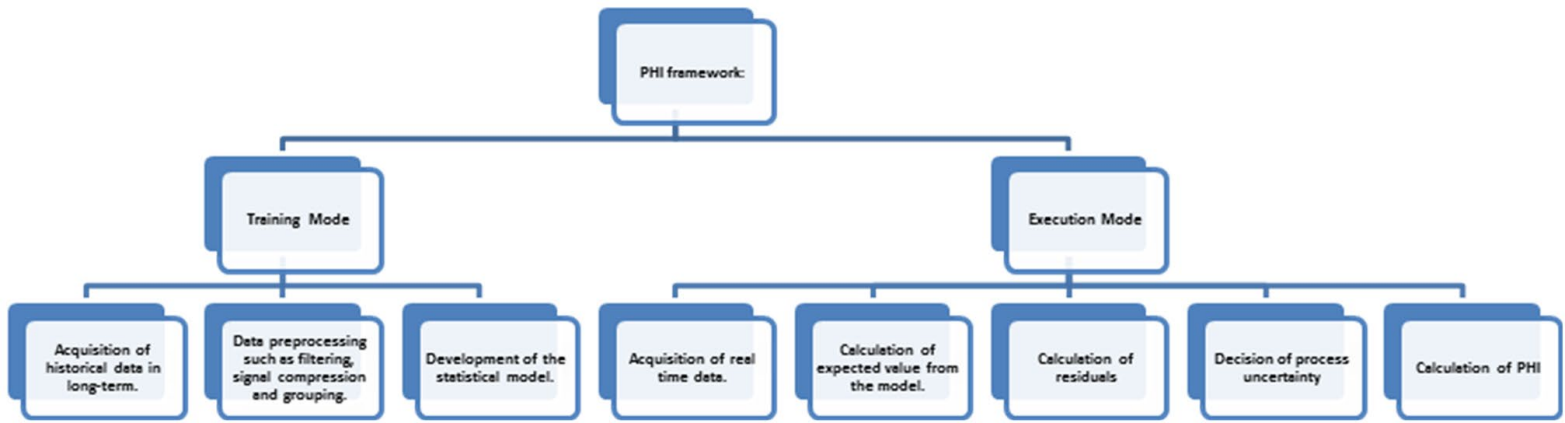
Evaluation of Health Index.

The framework of PHI is shown in Fig. 10. The sequence of actions in the training mode is as follow:

1. Acquisition of historical data in the long term.
2. Data preprocessing such as filtering, signal compression, and grouping.
3. Development of the statistical model.

On the other hand, the sequence of actions in the execution mode is as follows:

1. Acquisition of real-time data.
2. Calculation of expected value from the model.
3. Calculation of residuals.
4. The decision of process uncertainty.
5. Calculation of PHI.

In the execution phase, first step is to gather real-time data from the sensor signals and compare this information with the model estimates. Based on the comparison, the residuals between the model estimates and the real time measurements are evaluated. These residuals are used to predict the abnormalities in the plant. Suppose that the online values are [11 12 13 9 15] and the model estimates [11 12 13 14 15], then the estimated residuals will be [0 0 0 5 0]. These values are used in evaluating the process uncertainty (healthiness) by applying Eq. (2). On the other hand, process margins refer to the differences between alarms/trips and the operational conditions, which are evaluated using Eq. (1). An early warning is generated when an abnormal process uncertainty is observed earlier than a process margin. The process margins and process uncertainties are combined in overall health indices using Eq. (3).

The PHI system has been developed using MATLAB. A modular approach has been used so that modifications may be easily introduced, and new algorithms may be added, integrated, and tested as independent modules. This approach was found quite appropriate for research and development purposes. Moreover, the PHI system is delivered as executable MATLAB files.

### Features and functionalities of PHI

The main features and functionalities of PHI are (1) detecting the process uncertainty, in terms of a health index, for individual signals as well as for an entire plant, (2) warning anomalies in health indices, and (3) customized user interfaces and historians. Furthermore, since the PHI separately deals with safety-related and performance-related health indices, users can have appropriate decision-making in terms of their situation.

### System architecture

**Figure 11:**
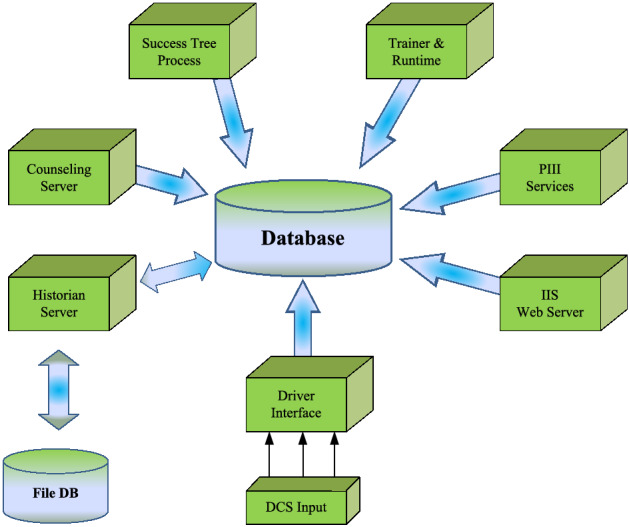
Server architecture of the PHI system ^26^.

**Figure 12:**
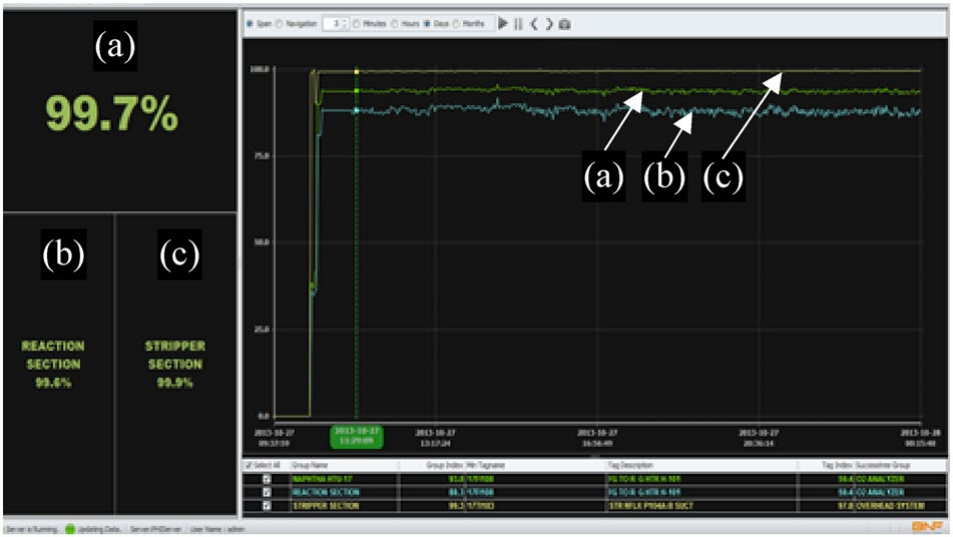
Example display of the PHI indicating the (**a**) overall plant health index and the health indices of the (**b**) reaction and (**c**) stripper sections.

PHI system is a client–server-based architecture, as shown in Fig. 11. The server side is divided into the core modules necessary to build the PHI functionality and PRISM, a real-time BNF (Breakthrough and Fusion) technology database. The clients are divided into the standard client and the web-based client. Figure 12 shows the main display of the PHI client. All of these functions bridge the information of the server-side with users.

The results of the PHI can be monitored through the client computer, which has the following main features:

1. Index display: the default display shows the index in percent of the topmost groups, including the trend. The index of other subsystems can be seen and accessed as well.
2. Success tree display: The success tree display having a hierarchical display and the group-wise display.
3. Trend display: A trend display showing the actual-expected value trend.
4. Alarms display: A grid-based alarm display showing the latest alarm on the top display.
5. Reports: Reports can be generated about the health status and regular alarm.
6. Configuration Manager: A configuration manager, which invokes at the beginning of the PHI Client application. The configuration manager checks for the port and the server’s IP address; if not able to connect, the configuration manager window will pop up at the startup.

## Selected refinery process

The process section selected for the implementation of the PHI system is a Hydrotreating Unit, which consists of a reaction section having two catalytic reactors in series and a stripper section.

**Figure 13:**
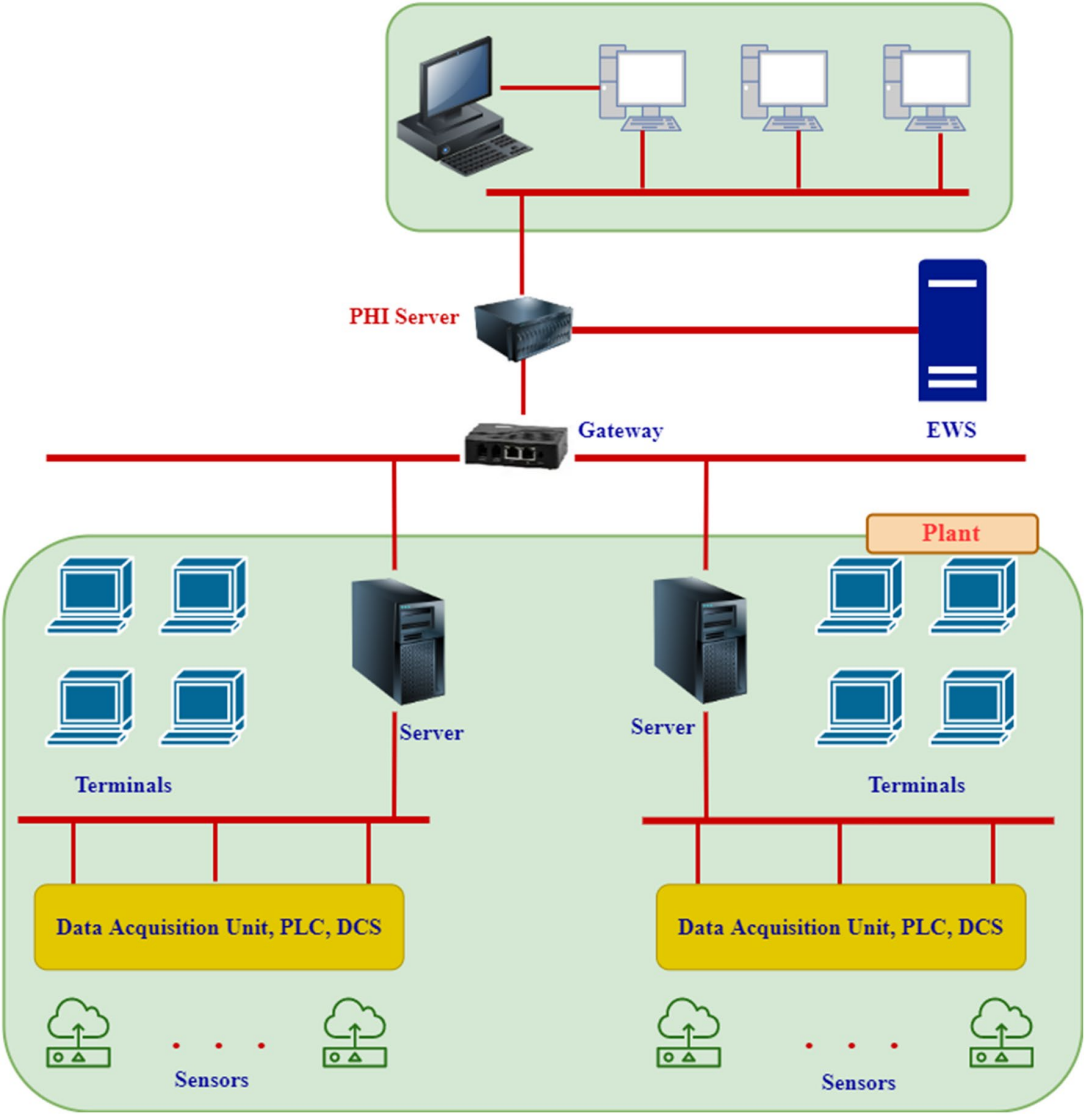
PHI System implementation architecture.

PHI system architecture is described in Fig. 13. System specifications can be summarized as follows:

**Table:** 

Interfacing:	Direct or through Open Platform Communications (OPC)
Server/Client:	MS Windows based
Implementation:	Fleet-wise, plant-wise & component wise
Typical Implementation Duration:	3 ~ 4 Weeks /Unit
Data communication:	Read only
Redundancy/ Hi-availability:	Available
Historian:	Built-in

**Figure 14:**
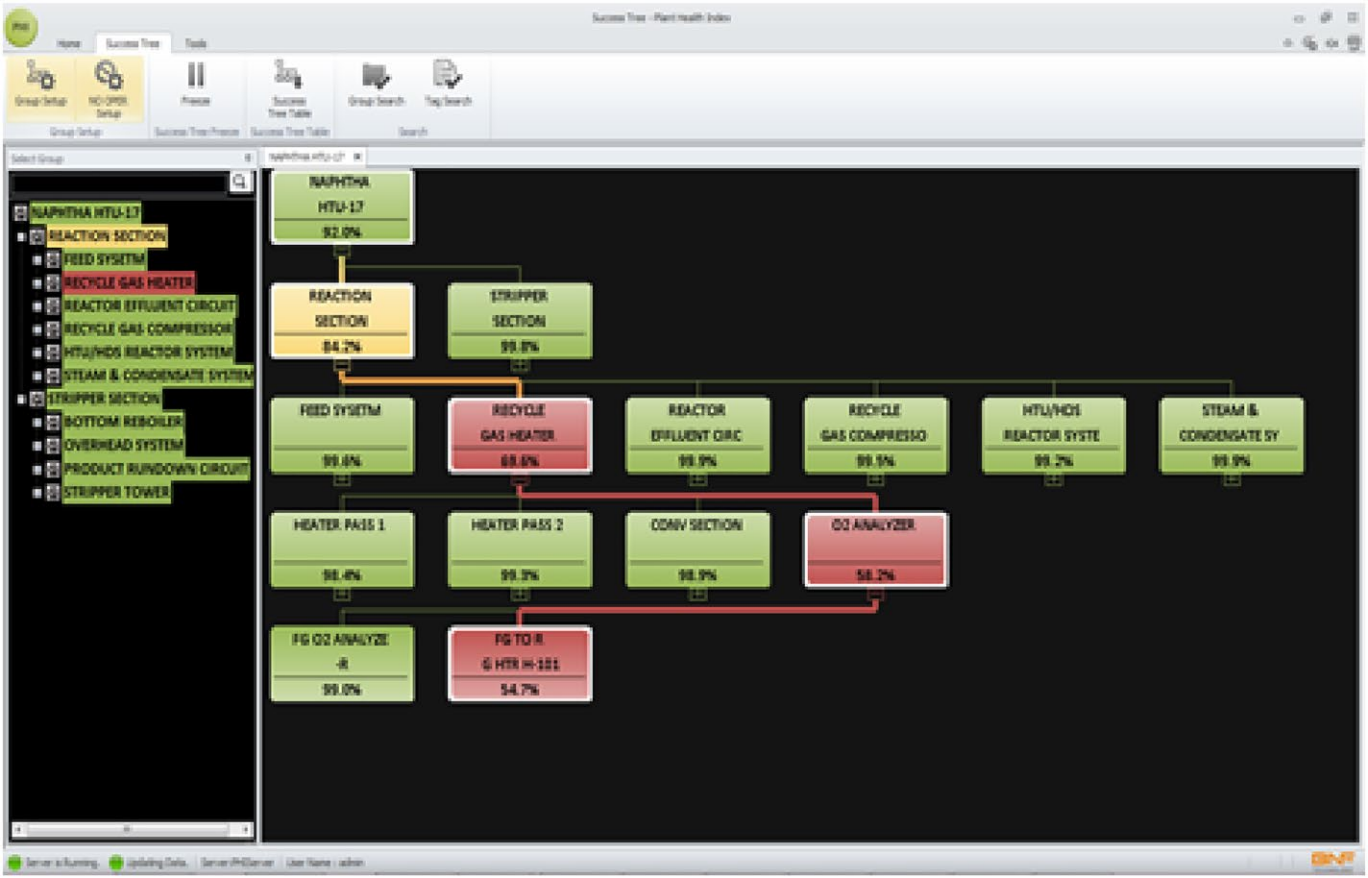
Success tree for the Hydrotreating unit.

The hierarchical representation of the plant under study in terms of groups and tags is known as success tree, as shown in Fig. 14. The groups are formed with the main system and the subsystems to be observed. Modifications, deletions, and additions to the groups are all possible. There is no restriction on the count of groups, but the number of components (children) that can be accommodated by each group is restricted to 10 subgroups or tags. The success tree initializes the weights of groups and tags. It receives data about groups and tags from a database, acquires tag index info from a PHI image, and generates group index on a regular basis. The refinery hydrotreating unit was divided into two main subsystems: reactor and stripper. Furthermore, six subsystems were defined for the reactor system and four were defined for the stripper system. The total number of components (tags) that were associated to the hydrotreating unit is 170. Twelve months data records were collected for all tags.

## Results and discussion

PHI has been installed, operated, and tested in the selected refinery hydrotreating unit. Besides indicating the healthiness of the plant, PHI provided a convenient solution for monitoring and detecting operational malfunctions. Operators, process engineers, supervisors as well as team leaders and managers found the system a stress-free and flexible tool for detecting faults and avoiding alarms. In addition, it provided an environment for enhancing daily operational practices and understanding the root causes of detected faults. These benefits were observed while discussing the case studies with operators and process engineers. Based on their experience in operating the process, their contributions were vital in explaining the symptoms and detected deviations.

For team leaders and managers, PHI enabled them to check operational wellness at a glance. They indicated that the system would give them an early warning and enough time for proper diagnosis and precise decisions. In case a decline in the overall index is observed, PHI will enable them to identify the root cause and contact the proper department to interfere and get back the index to its normal value.

The main challenge faced while implementing PHI is convincing the operators to check it and explore its functionalities. To overcome this challenge, it was recommended that supervisors and team leaders check the PHI system for 15 to 30 min every morning. Communication was then established at different levels to find solutions to observed abnormalities and introduce corrective actions.

Another observed benefit is the detection of a number of faulty signals. Some of these signals were not significant for the operation of the selected process, while several other signals were critical for the operation and were accordingly attended and corrected.

Other benefits that have been realized are as follows:

1. Convenient and easy way to monitor the operational conditions, specifically for chief operators, process engineers, and managers.
2. Reducing the time needed to identify the problem during operational abnormality or malfunction.
3. Increased reliability and a positive learning experience for the operators.
4. Friendly graphical user interface that both technical and non-technical staff may use.
5. Ability to navigate through the system’s hierarchical model (success tree) to trace the exact location of the potential problem.
6. Simple and convenient presentation of related plant tags.
7. Ability to trace and visualize historical data, as well as displaying and comparing selected tags.
8. A convenient way to monitor selected important signals.
9. Faulty signals can be easily detected.
10. Flexibility in displaying and comparing both actual and expected values.
11. Indicates clearly that a major malfunction of any part of the plant is always proceeded by small spike-shaped upset days before.
12. Good confidence with PHI statistical model which makes adding physical knowledge on the model unnecessary.

### Case studies

The performance of PHI in monitoring the selected hydrotreating unit was studied by analyzing the online measurements and data for a period of five months. The index dropped many times to very low values but in general, the reactor sub-index was lower than the stripper sub-index, indicating that the reactor section is causing most of the problems in the hydrotreating unit. Indication of malfunction occurrences is taken more seriously when time plots of the tags take curved shapes that span longer durations than short spike changes.

Two case studies have been selected for demonstration purposes and will be presented and discussed in this section. For each case study, symptoms will be first identified, followed by deducing the main causes for the defined abnormality. Conclusions are then derived from the analysis of the symptoms and deviations from nominal conditions. Moreover, snapshots of PHI screens are presented to illustrate the practical procedure followed in identifying and analyzing anomalies.

#### Case study one

**Figure 15:**
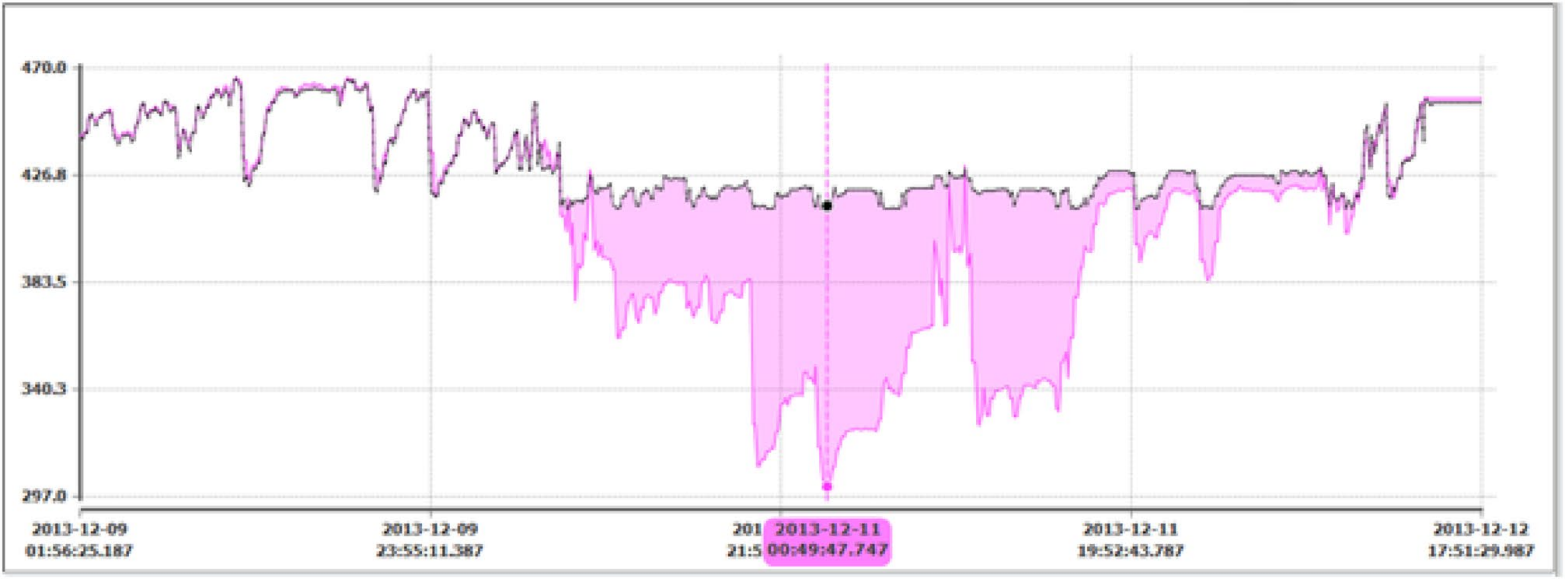
Case 1. Drop in steam temperature. Comparison of the actual and expected trends.

*Symptoms*: For this case study, PHI indicated deviation in a tag related to steam temperature. For this instance, the steam temperature was reduced to 113°F below the normal expected value. A screen snapshot showing the actual and expected values is shown in Fig. 15. The deviation lasted for nearly two days.

**Figure 16:**
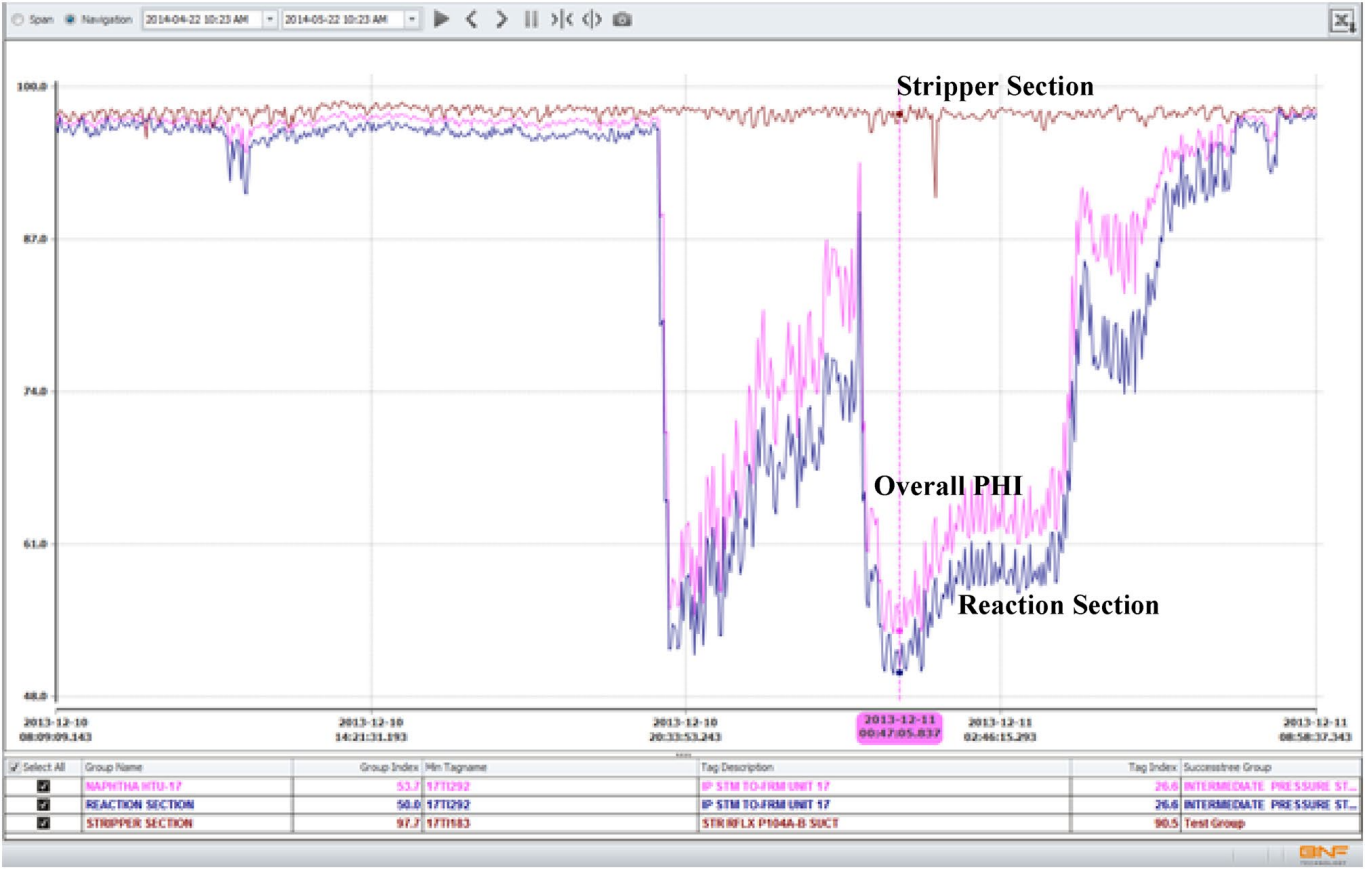
Case 1. Drop in steam temperature caused the drop in the health index of the reaction section and resulted in dropping the PHI to 53.7%.

As shown in Fig. 16, the abnormal conditions caused by the steam temperature resulted in dropping the overall plant health index to 53.7%. Moreover, the individual index value for the steam temperature tag dropped to 26.6%.

*Cause*: Normally the exhaust steam of the recycle compressor is at 150 psi, and as compared to the refinery header steam (IP), the temperature is high. Hence, for lowering this high temperature to the refinery header temperature, a de-super header is supplied with a boiler feed water injection system. Further analysis resulted in identifying a local temperature controller that have malfunctioned during this time. This controller failed in lowering the header temperature, caused model mismatch and resulted in dropping the overall plant health index.

*Case Conclusion*: This malfunction has been successfully detected by PHI and resulted in dropping the health index. Operators of the unit appreciated the ability of PHI to detect the abnormality. They considered the case as an important finding because malfunctioning of the local controller resulted in water hammering at the utility header. They stated that the present Distributed Control System (DCS) does not have the facility to monitor this effect. This is a significant finding by PHI and provides good evidence that monitoring the health index enables the operators to take early actions to prevent damages or even alarms.

#### CASE study two

**Figure 17:**
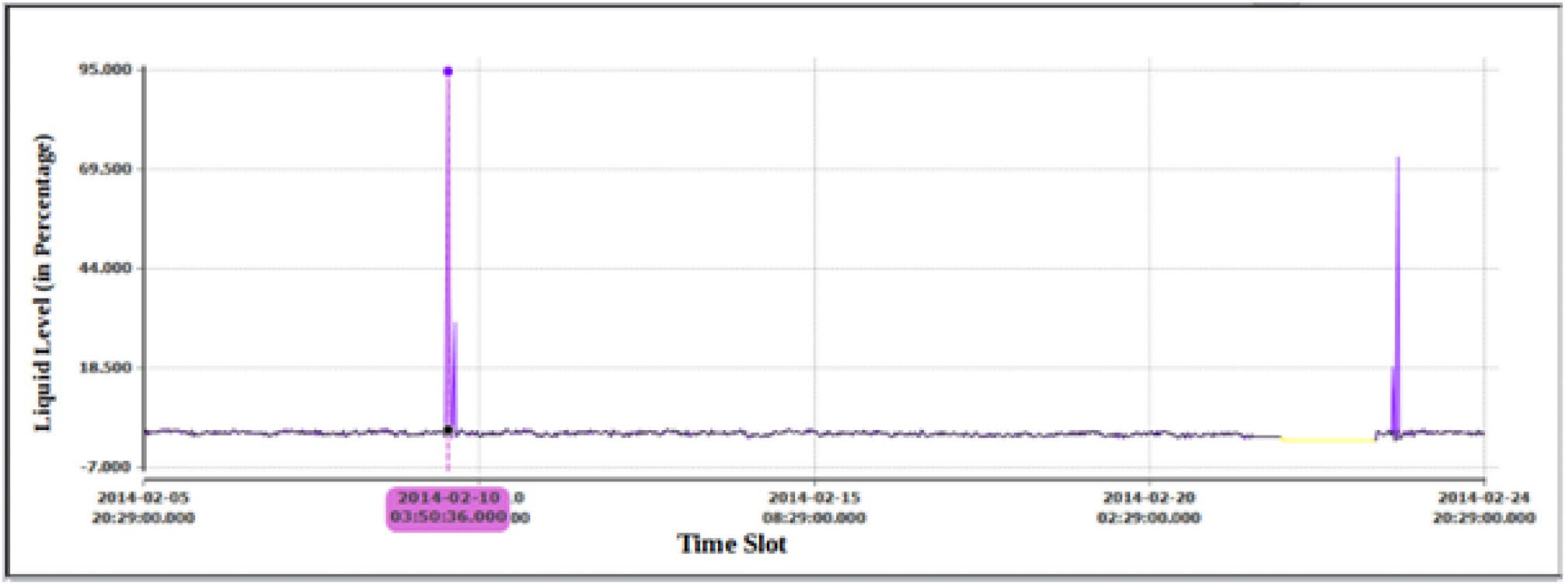
Case 2. sudden increase in liquid level of the bottom of the vaporizer.

**Figure 18:**
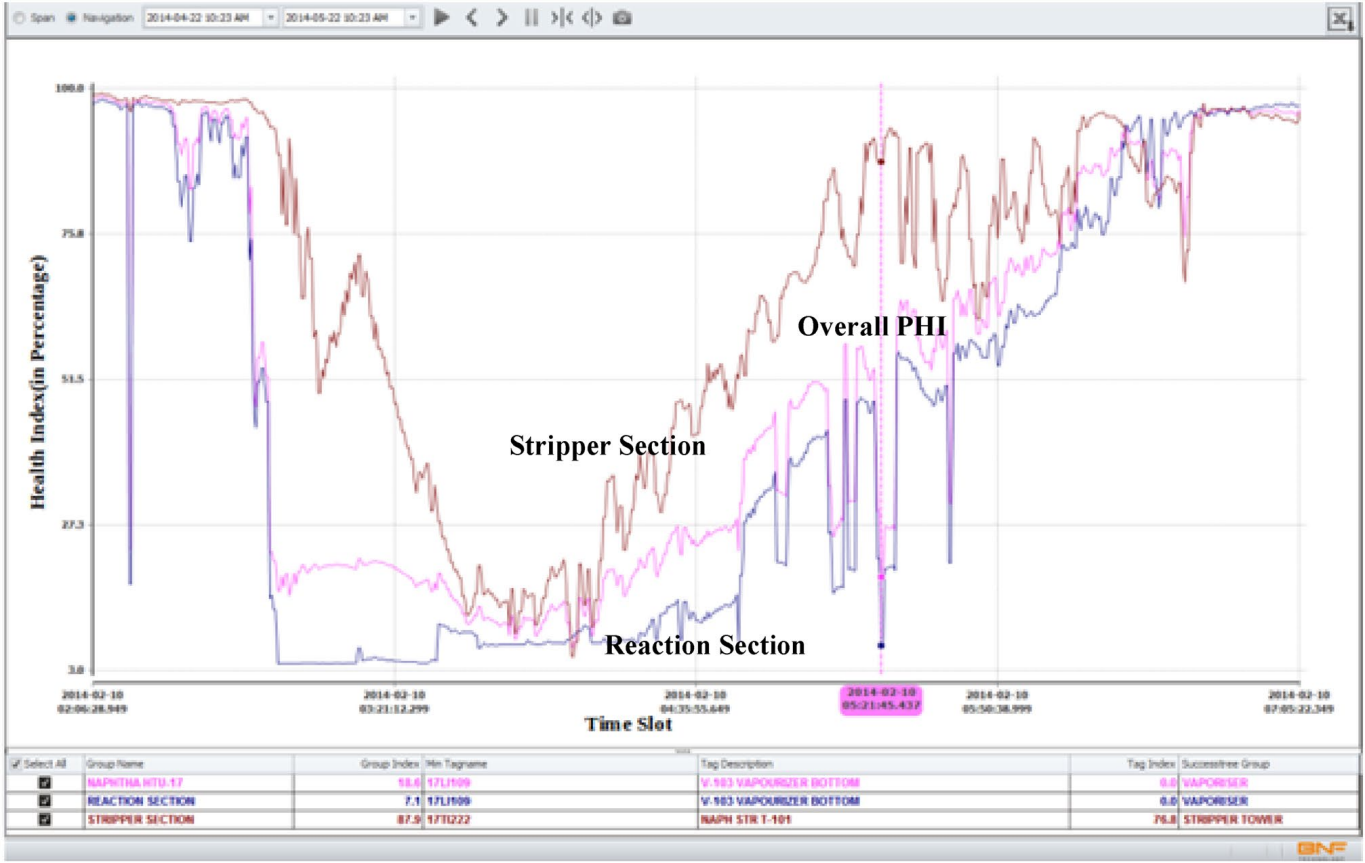
Case 2. Sudden increase in the liquid level resulted in dropping the overall PHI as well as the health indices related to the reaction and stripper sections.

*Symptoms*: For this case study, PHI indicated deviation in a tag related to the liquid level at the bottom of the vaporizer. In this deviation, the liquid level in the bottom of the vaporizer was increased to as high as 94.5% compared to the normal expected value of 2.7%. A screen snapshot showing the actual and expected values is shown in Fig. 17. The deviation lasted for two hours. As shown in Fig. 18, this abnormal condition caused by the increase in liquid level resulted in dropping the overall plant health index to 18.6%. In addition, the individual index value for this tag was dropped as low as 0.0%.

*Cause*: During this period, the unit was under startup condition. When the feedstock is heavy, it tends to collect liquid in drum. However, in Normal operation, no liquid collection is expected at those operating conditions. The data shows that during startup, reactor temperatures were picking up gradually; hence, the liquid accumulated in the drum and level went up.

*Case Conclusion*: The present Distributed Control System (DCS) has an adequate alarm system to observe this case. However, a PHI alarm would be an additional support to avoid the emergency condition due to abnormal level build-up in level, leading to reactor top flange leak if unattended.

At startup, so many actions are taken to get the plant at a steady state. In most cases, operators would be busy monitoring key tags and driving them to their desired values. Even though PHI was trained on the normal operation or steady-state data, it succeeded in identifying the abnormality discussed in this case study. Given this fact, the performance of PHI may be further enhanced to monitor the startup and shutdown procedures. In this case, the health index will provide better support to the operators in more difficult operational situations.

Based on the case study discussed above, a research and development study will be initiated to investigate the feasibility of incorporating the startup and shut down operations of process units. A pilot study will be carried out in which PHI will be trained on historical data related to startup and shut down. The main difficulty is that this task requires intensive process-related knowledge; hence, operators and process engineers should be involved in the study.

## Conclusions

A new technology using big data analytics to detect anomalies in process operation has been implemented and tested in this research work. Specifically, the Plant Health Index (PHI) applicability in identifying abnormalities in refinery operations has been investigated. PHI was applied on a Hydrotreating unit. The system performs online monitoring and compares online plant data with normal operating conditions, estimated using a nonparametric empirical model. The model was developed and tested.

The main benefit, which has been realized by the research team and the operators at the oil refinery, is the ability to use big data analytics to monitor and diagnose the performance of the unit by just watching the index value on screen. A considerably complex system was easily monitored, and root causes were also navigated systematically. An encouraging reason for using PHI is its ability to detect anomalies and provide early warnings before being detected by the Distributed Control System (DCS). The case studies demonstrated that operators might be alerted about possible upsets days before their occurrence.

For the upmost benefit, the unit operators recommended incorporating additional tags for better monitoring of the process. For example, additional tags may include those related to rotating equipment, such as compressors, to detect anomalies in pressure and vibration and include them in health index evaluation.

For earlier versions of PHI, evaluation of the health index was based on design ranges of the parameters. Application of PHI to the refinery process verified that historical ranges rather than design ranges are more practical and provide better predictions. Design ranges are usually wide and result in less sensitivity and precision. Approaching the limits of the nominal historical operating range is considered an early indication of malfunction while reaching the limits of the design range is in itself a state of failure.

In conclusion, the big data analytics approach proposed has been useful for exploring possible faults before they occur. This a good demonstration of employing data analytics in the oil refinery industry and building a successful big data strategy.

## Data Availability

The datasets used and/or analysed during the current study available from the corresponding author on reasonable request.
